# Different Subtypes of GABA-A Receptors Are Expressed in Human, Mouse and Rat T Lymphocytes

**DOI:** 10.1371/journal.pone.0042959

**Published:** 2012-08-21

**Authors:** Suresh K. Mendu, Amol Bhandage, Zhe Jin, Bryndis Birnir

**Affiliations:** Molecular Physiology and Neuroscience, Department of Neuroscience, Uppsala University, Uppsala, Sweden; University of Oslo, Norway

## Abstract

γ-aminobutyric acid (GABA) is the most prominent neuroinhibitory transmitter in the brain, where it activates neuronal GABA-A receptors (GABA-A channels) located at synapses and outside of synapses. The GABA-A receptors are primary targets of many clinically useful drugs. In recent years, GABA has been shown to act as an immunomodulatory molecule. We have examined in human, mouse and rat CD4^+^ and CD8^+^ T cells which subunit isoforms of the GABA-A channels are expressed. The channel physiology and drug specificity is dictated by the GABA-A receptor subtype, which in turn is determined by the subunit isoforms that make the channel. There were 5, 8 and 13 different GABA-A subunit isoforms identified in human, mouse and rat CD4^+^ and CD8^+^ T cells, respectively. Importantly, the γ2 subunit that imposes benzodiazepine sensitivity on the GABA-A receptors, was only detected in the mouse T cells. Immunoblots and immunocytochemistry showed abundant GABA-A channel proteins in the T cells from all three species. GABA-activated whole-cell transient and tonic currents were recorded. The currents were inhibited by picrotoxin, SR95531 and bicuculline, antagonists of GABA-A channels. Clearly, in both humans and rodents T cells, functional GABA-A channels are expressed but the subtypes vary. It is important to bear in mind the interspecies difference when selecting the appropriate animal models to study the physiological role and pharmacological properties of GABA-A channels in CD4^+^ and CD8^+^ T cells and when selecting drugs aimed at modulating the human T cells function.

## Introduction

The GABA-A receptor is an ion channel that opens when γ-aminobutyric acid (GABA) binds to its binding site on the receptor complex. In the brain, GABA is the most important neuroinhibitory transmitter. It is released from neuronal presynaptic terminals and activates the GABA-A channels located at the postsynaptic site [1]. GABA in low, submicromolar concentrations is also present around the neurons where it activates high-affinity GABA-A channels located outside of synapses [1], [2]. These channels are called extrasynaptic GABA-A channels [3]. Although the focus over the years has been on the GABA-A channels in the brain, there is growing evidence indicating a significant, physiological function of GABA and GABA-A channels in a number of non-neuronal tissue [4]. Components of the GABA signaling systems have also been identified in cells of the immune system [5], [6], [7], [8], [9], [10], [11], [12], [13].

The GABA-A ion channel is a pentameric chloride channel that is commonly made of three different types of subunits. To-date 19 different mammalian GABA-A subunits have been cloned (α1–6, β1–3, γ1–3, δ, ε, π, θ, ρ1–3) [14]. The subunits can combine in many different arrangements to form the pentameric channel. This is important as the specific subunits in the channel complex determine the pharmacological specificity of the channel [14]. It has been shown that benzodiazepine-site ligands can differentiate between GABA-A channel subtypes based on the type of α and γ subunits present in the channel complex [1]. Other drugs like the general anaesthetics and even GABA have been used to extend the list of subtypes further as their affinity is also related to the subunit composition of the channels [1]. In immune cells, the GABA-A channels are not located in a synapse and are only exposed to low concentrations of GABA similar to extrasynaptic GABA-A channels in neurons.

GABA is present in blood in submicromolar concentration [15], [16] and may be produced by the immune cells themselves [7], [17]. Immune cells such as CD4^+^, CD8^+^ T cells and macrophages do express GABA-A channels [6], [7], [9], [11], [18], [19] but what subtypes are present is generally not known. Only in two of the studies on immune cells has the expression of all 19 GABA-A subunits been examined and revealed that the subsets of GABA-A subunits varied between a mouse T cell line and rat T lymphocytes [8], [18] raising the question of interspecies difference in terms of GABA-A channel expression in T cells. The GABA signaling system is active in immune cells and appears to modulate a wide variety of functional properties of the cells including cell proliferation, cytokine secretion, phagocytic activity and chemotaxis [7], [8], [11], [13], [18], [19], [20]. As the functional and pharmacological properties are dictated by the pentameric subunit-composition of the GABA-A channel complex, it is important to know the subtypes in the cells when selecting drugs to act on the channels. A number of drugs modulating the functional properties of GABA-A channels are widely used in clinical settings. These drugs include the general anesthetics and benzodiazepines that differ in their selectivity at GABA-A channels. In order to minimize side-effects involving the immune system when these drugs are used, it is essential to know which GABA-A subtypes are formed in the immune cells. Furthermore, when selecting the appropriate experimental animal model, it is desirable that it expresses similar channel subtypes to their human counterpart. T lymphocytes have a central role in the cell-mediated immune response of the adaptive immune system. We have previously shown that 10 of the 19 GABA-A channel subunit mRNAs (α1, α2, α3, α4, α6, β3, γ1, δ, ρ1, ρ2) are expressed in both CD4^+^ and CD8^+^ T cells from biobreeding (BB) rats [18]. Here we examined the interspecies variability in terms of expression of the 19 GABA-A channel subunit isoforms in CD4^+^ and CD8^+^ T cells from humans, rats and mice. It is the first time all potential GABA-A channel subtypes in native T cells have been examined and compared for the three species. The results show that different combinations of GABA-A channel subunit isoforms are expressed in T cells from humans, rats and mice.

## Results

### Differential expression of GABA-A channel subunit mRNAs in human, rat and mouse CD4^+^ and CD8^+^ T cells and Jurkat cells

Here we examined whether the 19 different GABA-A channel subunit mRNAs were present in CD4^+^ and CD8^+^ T cells from rats (Wistar), mice (C57BL/6J) and humans as well as in a human CD4^+^ T cell line, the Jurkat cells. Primers specific for each GABA-A channel subunit (Table 1) were verified using the appropriate brain cDNA samples from humans, rats or mice.

**Table 1:** Human, rat and mouse primers list for quantitative real-time RT-PCR.

Gene name	Forward primer (5′-3′)	Reverse primer (5′- 3′)	Product size (bp)
Human			
α1 (*GABRA1*)	GGATTGGGAGAGCGTGTAACC	TGAAACGGGTCCGAAACTG	66
α2 (*GABRA2*)	GTTCAAGCTGAATGCCCAAT	ACCTAGAGCCATCAGGAGCA	160
α3 (*GABRA3*)	CAACTTGTTTCAGTTCATTCATCCTT	CTTGTTTGTGTGATTATCATCTTCTTAGG	102
α4 (*GABRA4*)	TTGGGGGTCCTGTTACAGAAG	TCTGCCTGAAGAACACATCCA	105
α5 (*GABRA5*)	CTTCTCGGCGCTGATAGAGT	CGCTTTTTCTTGATCTTGGC	105
α6 (*GABRA6*)	ACCCACAGTGACAATATCAAAAGC	GGAGTCAGGATGCAAAACAATCT	67
β1 (*GABRB1*)	CCAGGTCGACGCCCACGGTA	GTGGCCTTGGGGTCGCTCAC	102
β2 (*GABRB2*)	GCAGAGTGTCAATGACCCTAGT	TGGCAATGTCAATGTTCATCCC	137
β3 (*GABRB3*)	CCGTTCAAAGAGCGAAAGCAACCG	TCGCCAATGCCGCCTGAGAC	105
γ1 (*GABRG1*)	CCTTTTCTTCTGCGGAGTCAA	CATCTGCCTTATCAACACAGTTTCC	91
γ2 (*GABRG2*)	CACAGAAAATGACGGTGTGG	TCACCCTCAGGAACTTTTGG	136
γ2 (*GABRG2)-2* *	AGCAACCGGAAACCAAGC	TCCATTTTGGCAATGCGG	269/245
γ2 (*GABRG2)-3*	AACATGGTGGGGAAAATCTG	GGCAGGAGTGTTCATCCATT	196
ε (*GABRE*)	TGGATTCTCACTCTTGCCCTCTA	GGAGTTCTTCTCATTGATTTCAAGCT	107
θ (*GABRQ*)	CCAGGGTGACAATTGGCTTAA	CCCGCAGATGTGAGTCGAT	63
π (*GABRP*)	GGCCTTGCTAGAATATGCAGTTG	CTTTGTTGTCCCCCTATCTTTGG	76
ρ1 (*GABRR1*)	Hs00266687_m1 from AppliedBiosystem	Hs00266687_m1 from AppliedBiosystem	94
ρ2 (*GABRR2*)	CCTAGAAGAGGGCATAGACATCG	TCCAGTAGCTGCTGCATTGTTTG	99
ρ3 (*GABRR3*)	TGATGCTTTCATGGGTTTCA	CGCTCACAGCAGTGATGATT	111
*B2M*	CCTGCCGTGTGAACCATGTGACT	GCGGCATCTTCAAACCTCCATGATG	94
Rat			
α1 (*Gabra1*)	CTCCTACAGCAACCAGCTATACCC	GCGGTTTTGTCTCAGGCTTGAC	113
α2 (*Gabra2*)	AAGAGAAAGGCTCCGTCATG	GCTTCTTGTTTGGTTCTGGAGTAG	134
α3 (*Gabra3*)	ACAAGCACCACCTTCAACATAG	AGGTCTTGGTCTCAGCAGGA	174
α4 (*Gabra4*)	GATGTCAACAGCAGAACTGAGGTG	TTGTGCCAGATCCAGAAGGTGGTG	345
α5 (*Gabra5*)	GCCTTGGAAGCAGCTAAAATC	GAAGTCTTCTCCTCAGATGCTCT	178
α6 (*Gabra6*)	CACTCTGACTCCAAGTACCATCTG	GTACACAAGGTTGAATCCTG	221
β1 (*Gabrb1*)	CCCTCAGAAAAAAGGAGCGA	TCACGGCTGCTCAGTGGTTT	231
β2 (*Gabrb2*)	GCCTGGATGTCAACAAGATGGACC	CTAGGCAACCCAGCTTTCCGATAC	169
β3 (*Gabrb3*)	CCTACTAGCACCGATGGATGTT	GATGCTTCTGTCTCCCATGTAC	163
γ1 (*Gabrg1*)	TGACACGTTCTTCAGGAACTCAA	AACCCTTCCATCACTCCATATCC	91
γ2 (*Gabrg2*)	CGGAAACCAAGCAAGGAT	TCTCTTGAAGGTGGGTGGCA	134
γ2 *(Gabrg2)-2*	CGGCCCGACATAGGAGTGAAACC	TCGTGATCCAGTGAGCATCCGC	250
γ2 (*Gabrg2)-3*	CGCTCTACCCAGGCTTCACTAGC	TCGGGCCGAAGTTTGTTGTCGT	157
ε (*Gabre*)	CAGATGGCTCTCATCCATAAGGA	GCTAGAGAAAGACAGAGGGCAAGA	129
θ (*Gabrq*)	TAGGACTTGGCTGGCAGAGAGTA	ATGCTGGAGGAGAGCTCGAA	72
π (*Gabrp*)	TGACAACAGTGTTGTCCATGACA	TGGCCTTTATGAAGCAGTTGGT	81
ρ1 (*Gabrr1*)	TGGACAGCAGCTACAGTCACGG	AAGCAGCTGGGAAAATGATC	209
ρ2 (*Gabrr2*)	CAAGAAGCCACATTCTTCCA	TTCTGGAAGATATAGAGTCC	133
ρ3 (*Gabrr3*)	GGTGTGAGCGCCTCTATGC	GGGAGCTGACCCACATGTACA	70
*Hprt*	CTCATGGACTGATTATGGACAGGAC	GCAGGTCAGCAAAGAACTTATAGCC	123
Mouse			
α1 (*Gabra1*)	AAA AGC GTG GTT CCA GAA AA	GCT GGT TGC TGT AGG AGC AT	84
α2 (*Gabra2*)	GCTACGCTTACACAACCTCAGA	GACTGGCCCAGCAAATCATACT	117
α3 (*Gabra3*)	GCCGTCTGTTATGCCTTTGTATTT	TTCTTCATCTCCAGGGCCTCT	118
α4 (*Gabra4*)	AGAACTCAAAGGACGAGAAATTGT	TTCACTTCTGTAACAGGACCCC	118
α5 (*Gabra5*)	GATTGTGTTCCCCATCTTGTTTGGC	TTACTTTGGAGAGGTGGCCCCTTTT	100
α6 (*Gabra6*)	GGTGACCGGGCATCCCAGTGA	TGTTACAGCACCCCCAAATCCTGGC	197
β1 (*Gabrb1*)	GGTTTGTTGTGCACACAGCTCC	ATGCTGGCGACATCGATCCGC	153
β2 (*Gabrb2*)	GCTGGTGAGGAAATCTCGGTCCC	CATGCGCACGGCGTACCAAA	70
β3 (*Gabrb3*)	GAGCGTAAACGACCCCGGGAA	GGGACCCCCGAAGTCGGGTCT	100
γ1 (*Gabrg1*)	ATCCACTCTCATTCCCATGAACAGC	ACAGAAAAAGCTAGTACAGTCTTTGC	100
γ2 (*Gabrg2*)	ACTTCTGGTGACTATGTGGTGAT	GGCAGGAACAGCATCCTTATTG	147
γ3 (*Gabrg3*)	ATTACATCCAGATTCCACAAGATG	CAC AGG TGT CCT CAA ATT CCT	149
δ (*Gabrd*)	TCAAATCGGCTGGCCAGTTCCC	GCACGGCTGCCTGGCTAATCC	147
ε (*Gabre*)	ACTGCGCCCTGGCATTGGAG	AGGCCCGAGGCTGTTGACAA	70
θ (*Gabrq*)	GCTGGAGGTGGAGAGCTATGGCT	CCCCAGGTACGTGTACTGAGGGA	115
π (*Gabrp*)	TCGGTGGTGACCCAGTTCGGAT	TCTGTCCAACGCTGCCGGAG	115
π (*Gabrp*)	TCGGTGGTGACCCAGTTCGGAT	TCTGTCCAACGCTGCCGGAG	115
ρ1 (*Gabrr1*)	CCATCTAGGAAAGGCAGCAG	GAGCTTCGTCTCAGGATTGG	92
ρ2 (*Gabrr2*)	GCTGCCTGTTGCATCATAGA	ATACAAATGGCTTGGCTTGG	153
ρ3 (*Gabrr3*)	CAACTCAACAGGAGGGGAAA	TCCACATCAGTCTCGCTGTC	101
β-actin(*Actb*)	GTCCACACCCGCCACCAGTTCG	ATGCCGGAGCCGTTGTCGAC	71

*B2M*: beta-2 microglobulin; *Hprt*: hypoxanthine guanine phosphoribosyl transferase.

* Primer sequences were extracted from Alam S, et al. (2006) Mol Immunol, 43(9): 1432-42.

**Figure 1:**
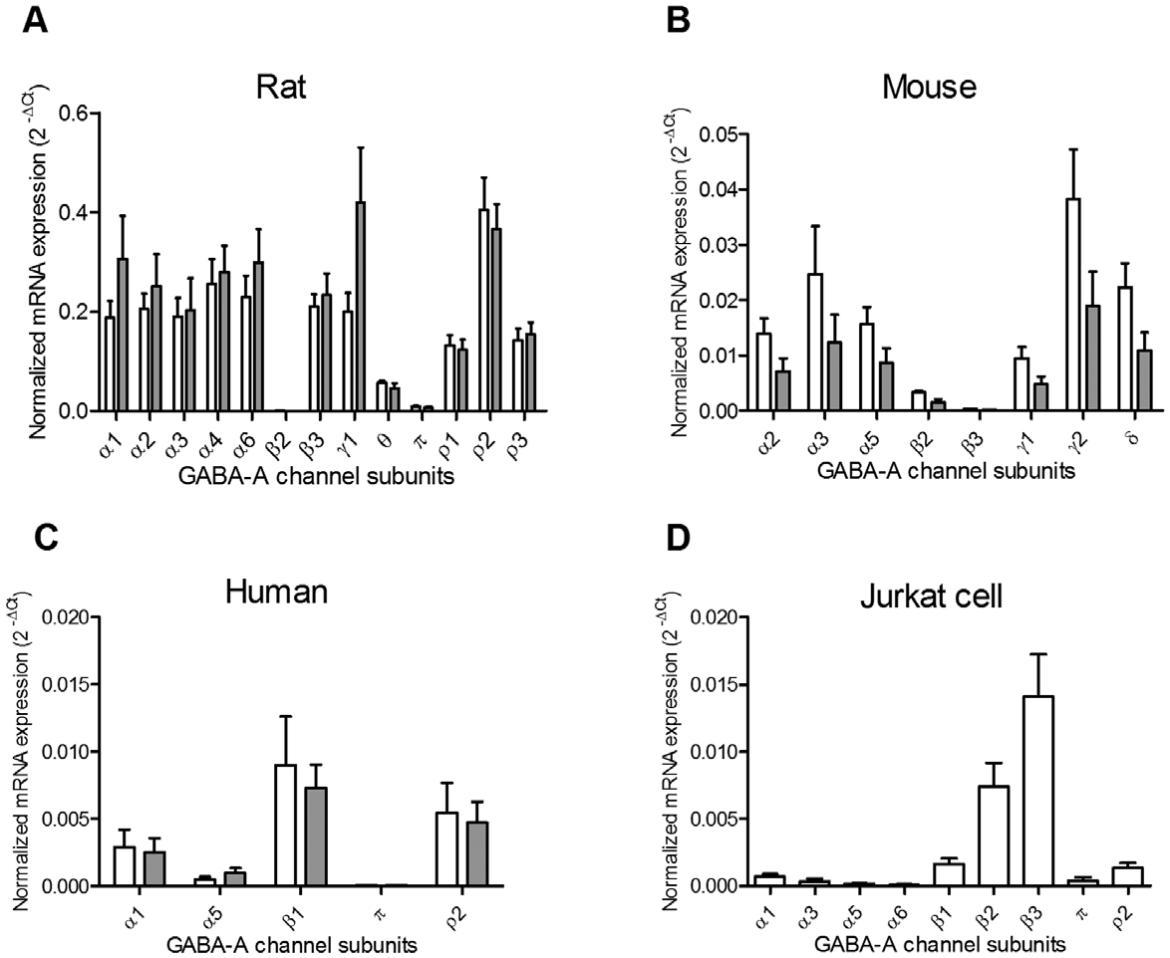
Expression of GABA-A channel subunit mRNAs in CD4^+^ or CD8^+^ T cells from rats, mice and human donors and the Jurkat cell line. Expression of all 19 GABA-A receptor channel subunit isoforms was examined using RT-qPCR in T cells. Subunit isoforms that were detected are shown in A–D. In CD4^+^ (open bar) and CD8^+^ (grey bar) T cells from mesenteric lymph nodes of Wistar rats (A, n = 4) or C57BL/6J mice (B, n = 3), 13 and 8 different GABA-A channel subunit mRNAs were detected, respectively. The mRNA expression level for each subunit did not differ between the CD4^+^ and the CD8^+^ T cells. (C) In both CD4^+^ (open bar) and CD8^+^ (grey bar) T cells isolated from human pancreatic lymph nodes (4 different donors) 5 different GABA-A channel subunit mRNAs were detected. (D) In Jukart cells, 9 different GABA-A channel subunit mRNAs were detected. The mRNA level of each subunit was normalized to reference genes, calculated as 2^−ΔCt^ and presented as mean with SEM. The reference genes were hypoxanthine phophoribosyltransferase (*Hprt*) for rat T cells, β-actin (*Actb)* for mouse T cells, and β2-microglobin (*B2M*) for human T cells and Jurkat cells.

In both CD4^+^ and CD8^+^ T cells isolated from mesenteric lymph nodes from Wistar rats, 13 GABA-A channel subunit mRNAs were detected (Fig. 1A and Table 2). There was an abundant expression of the α1, α2, α3, α4, α6, β3, γ1 and ρ2 subunits, modest expression of the θ, ρ1 and ρ3 subunits and low expression of the β2 and π subunits. The mRNA levels of these 13 GABA-A channel subunits did not differ between the CD4^+^ and CD8^+^ T cells (Fig. 1A).

**Table 2:** GABA-A channel subunit mRNA expression in rat, mouse and human T cells (both CD4^+^ and CD8^+^) and Jurkat cells.

GABA-A subunits	Rat	Mouse	Human	Jurkat cells
α1	++	−	++	+
α2	++	++	−	−
α3	++	++	−	+
α4	++	−	−	−
α5	−	++	+	(+)
α6	++	−	−	(+)
β1	−	−	++	++
β2	(+)	+	−	++
β3	++	(+)	−	++
γ1	++	+	−	−
γ2	−	++	−	−
γ3	−	−	−	−
δ	−	++	−	−
ε	−	−	−	−
θ	+	−	−	−
π	(+)	−	(+)	(+)
ρ1	+	−	−	−
ρ2	++	−	++	+
ρ3	+	−	−	−

++: Abundant expression; +: Modest expression; (+): Low expression: −: No expression.

In CD4^+^ and CD8^+^ T cells isolated from mesenteric lymph nodes from C57BL/6J mice (Fig. 1B and Table 2), 8 GABA-A channel subunit mRNAs were consistently detected in both cell types. There was abundant expression of the α2, α3, α5, γ2 and δ subunits, modest expression of the β2 and γ1 subunits and low expression of the β3 subunit. The mRNA levels of these 8 subunits did not differ between the CD4^+^ and CD8^+^ T cells (Fig. 1B).

Interestingly, we detected only 5 GABA-A channel subunit mRNAs in both CD4^+^ and CD8^+^ T cells isolated from human pancreatic lymph nodes (Fig. 1C and Table 2). These were the α1, α5, β1, π and ρ2 subunits and the mRNA levels did not differ between the CD4^+^ and CD8+ T cells (Fig. 1C). Using the same human GABA-A primer sets and reference gene (Table 1), 9 GABA-A channels subunit mRNAs including the α1, α3, α5, α6, β1, β2, β3, π and ρ2 subunits were detected in Jurkat cells, the human T cell lymphoblast-like cell line (Fig. 1D and Table. 2). The mRNA expression levels for the β1 and ρ2 subunits in Jurkat cells were significantly lower than those in the CD4^+^ or CD8^+^ human T cells (Kruskal-Wallis one-way ANOVA on ranks, post hoc Tukey test, p<0.05)

The γ2 subunit confers specific pharmacology on the GABA-A channel complex and was only detected in samples from the mouse T cells where it was abundantly expressed (Fig. 1B). We, therefore, designed additional primer pairs specific for the rat or the human γ2 subunit (Table 1) to further examine whether the γ2 subunit could be detected in the rat and human T cells. These primers were designed to amplify all splice variants of γ2 transcript and targeted different regions of the γ2 transcript from those used for the quantitative PCR. These additional primers were also verified using the rat and human brain samples, respectively. However, no γ2 subunit mRNA transcript was detected in the rat and the human T cells or the Jurkat cells with these additional primer sets (data not shown).

### GABA-A channel subunit proteins in the native lymphocytes and the Jurkat cell line

**Figure 2:**
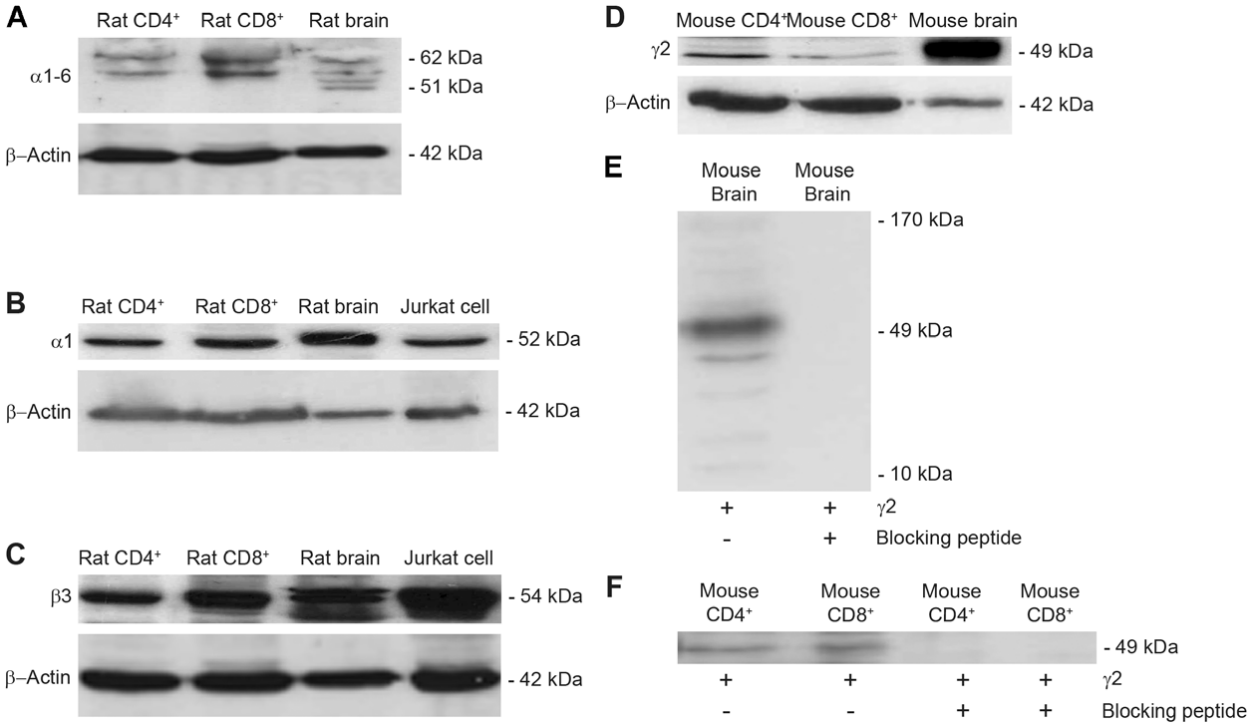
Detection of GABA-A channel subunit proteins in CD4^+^ and CD8^+^ T cells from rats, mice and the Jurkat cell line. (A–C). Rat CD4^+^ and CD8^+^ T cell protein extracts immunoprecipitated with anti-α1–6 (A, n = 6), anti-α1 (B, n = 3) or anti-β3 antibody (C, n = 3) and bands at the correct molecular weight were identified. The α1 and β3 GABA-A channel subunits proteins were also identified in extracts from Jurkat cells (B and C). (D) The γ2 GABA-A channel subunit protein was detected in mouse CD4^+^ and CD8^+^ T cells (n = 4). Protein extracts from rat and mouse brains served as positive controls. In A–D, the blots with β-actin served as loading controls. (E–F) The bands specific for γ2 subunit were absent in the presence of the γ2 blocking peptide in protein extracts from mouse brain (E) and mouse CD4+ and CD8+ T cells (F). The amounts of proteins loaded were: (A) 20 µg for all lanes; (B) 60 µg for rat CD4^+^, CD8^+^ T cells and Jurkat cells, 15 µg for rat brain; (C) 30 µg for all lanes; (D, E, F) 60 µg for mouse CD4^+^ and CD8^+^ T cells; 10 µg for mouse brain. Molecular weight in kDa is given in Table S1.

Western blot analysis was used to confirm the presence of the GABA-A channel subunit proteins in rat and mouse CD4^+^ and CD8^+^ T cells and in the Jurkat cell line (Fig. 2). Experiments where the primary antibody was omitted served as negative controls and resulted in no specific bands at the predicted molecular weights in the blots. Protein extracts from rat and mouse brains served as positive controls whereas β-actin served as a control for protein loading.

In rat CD4^+^ and CD8^+^ T cells, the mRNAs of several alpha subunits (α1, α2, α3, α4 and α6) were detected by RT-qPCR. Therefore, we first used an antibody, GABA-A α1–6, that is reported to recognize all GABA-A channels α isoforms of mouse, rat and human origins. This polyclonal antibody was raised against amino acids 157–456 near the C-terminus of the human GABA-A α1 protein. This region is highly conserved: in rat GABA-A α1 (100%), α2 (80%), α3 (71%), α4 (76%), α5 (71%) and α6 (55%) proteins. The predicted molecular weight for the rat α GABA-A subunits ranges from 51 to 62 kDa. In a Western blot several bands were detected within the expected molecular weight range using protein extracts from rat brain (4 bands), rat CD4^+^ (2 bands) and rat CD8^+^ (2 bands) T cells (Fig. 2A). Surprisingly only a faint band was detected in the brain sample in the expected molecular range for the α1 subunit, which is the most highly expressed α subunit in the brain. We, therefore, further examined the α1 isoform using an α1-specific antibody that was raised against amino acids 28–43 of the rat GABA-A α1 protein. This antigen peptide sequence is also conserved in the human GABA-A α1 protein. Fig. 2B shows that a single band was detected at the expected molecular weight of ∼52 kDa in protein extracts from rat CD4^+^, CD8^+^ T cells and Jurkat cells as well as in protein extracts from rat brain that served as a positive control. However, some nonspecific bands were observed in the blot as well (data not shown).

The GABA-A β3 subunit mRNA was the most abundant β subunit isoform in rat CD4^+^ and CD8^+^ T cells as determined by the RT-qPCR. By using a GABA-A β3 antibody, a single band was detected at the expected molecular weight of ∼54 kDa in protein extracts from rat CD4^+^, CD8^+^ T cells and Jurkat cells (Fig. 2C). The molecular weight of the three different β subunits ranges from 54.1 to 54.6 kDa and all β-subunit antibodies available to-date show some cross reactivity for the three isoforms. In the protein extracts from rat brain there were 2 bands (∼54 kDa), which may indicate different β subunits isoforms, different post-translational modification or splice variants creating different size proteins. Some nonspecific bands were observed (data not shown).

The GABA-A γ2 subunit mRNA was detected only in mouse T cells. To examine whether the GABA-A γ2 subunit protein is present in mouse T cells, a GABA-A γ2 specific antibody raised against amino acids 39–67 of the mouse GABA-A γ2 protein was used. This antigen peptide sequence is also 100% conserved in the human and rat GABA-A γ2 protein. Fig. 2 shows that a single major band was detected at the molecular weight of ∼49 kDa in protein extracts from mouse brain (Fig. 2D and E) and mouse CD4^+^ and CD8^+^ T cells (Fig. 2D, E) but some minor nonspecific bands could also be detected (Fig. 2E). The predicted molecular weight of mouse GABA-A γ2 protein is 54 kDa, however, the cleavage of the signal peptide (the first 38 amino acids) leads to a 49 kDa γ2 protein. The specific band was abolished by pre-absorption of the antibody with the synthetic GABA-A γ2 immunogenic peptide (Fig. 2E and F).

### Localization of GABA-A subunit proteins in native T lymphocytes and the Jurkat cell line

The Western blot results confirmed the presence of several GABA-A subunit proteins in T cells and Jurkat cells. We further examined the cellular localization of the GABA-A subunits by immunocytochemistry. Negative controls where the primary antibody was omitted were devoid of immunostaining (data not shown).

**Figure 3:**
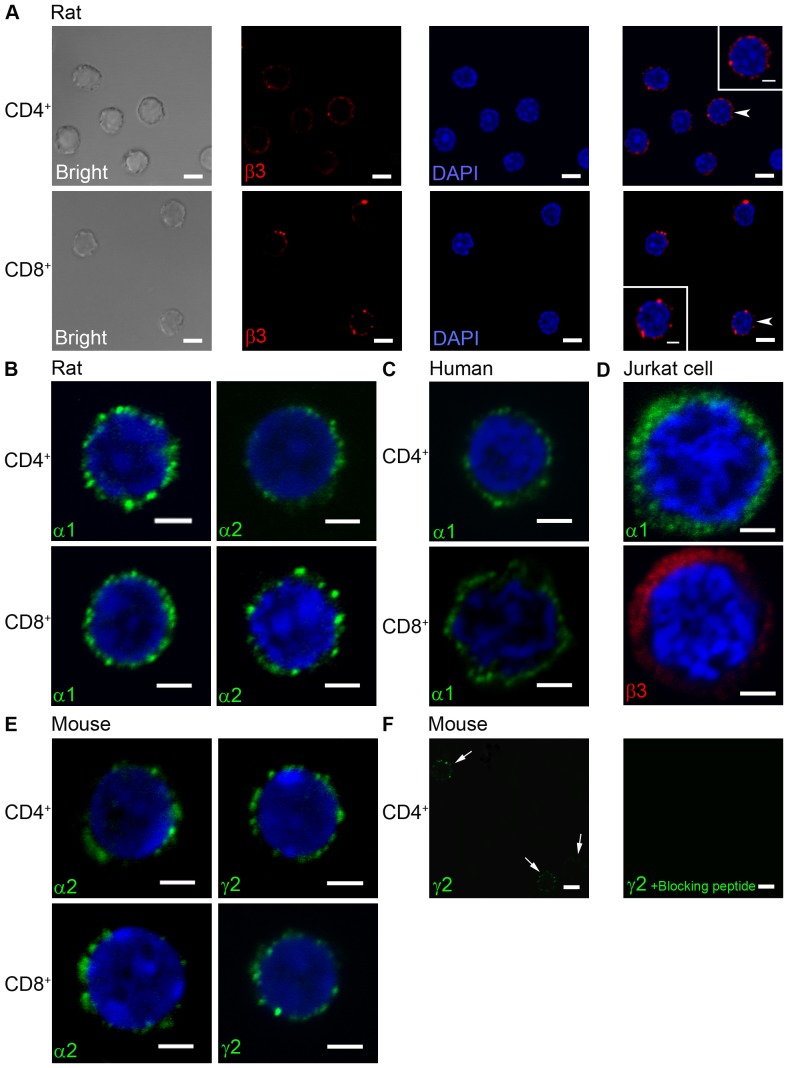
Immunolabeling of GABA-A channel subunits in rats, mice and human T cells and the Jurkat cell line. (A) Rat CD4^+^ (n = 85) and CD8^+^ (n = 186) T cells, β3 GABA-A channel subunit immunolabeling is observed in most cells. Insert in (A) represent the cell identified with the arrowhead. (B) Rat CD4^+^ and CD8^+^ T cells, α1 (n = 44; n = 43), α2 (n = 53; n = 55) GABA-A subunit immunolabeled. The representative images show the punctate labeling pattern (C) Human CD4^+^ (n = 34) and CD8^+^ (n = 43) T cells, α1 GABA-A subunit immunolabeled. (D) Jurkat cells, α1 (n = 104) or β3 (n = 65) GABA-A subunit immunolabeled. (E) Mouse CD4^+^ and CD8^+^ T cells, α2 (n = 42; n = 53) or γ2 (n = 36; n = 54) GABA-A subunit immunolabeled. (F) In mouse CD4^+^ T cells, the γ2 GABA-A subunit immunolabeling was absent in the presence of the blocking peptide (n = 35). Subunit colour-labeling: α1 green; α2 green, β3 red; γ2 green. The nuclei were stained with DAPI (blue). Scale bars in A and F = 5 µm, in insert in A and in B–E = 2 µm.

In rat CD4^+^ and CD8^+^ T cells somewhat punctate immunofluorescent staining of the GABA-A α1, α2 and β subunit was observed (Fig. 3A and 3B). Similar staining patterns were observed for the GABA-A α1 subunit in human CD4^+^ and CD8^+^ T cells (Fig. 3C) and for the GABA-A α2 and the γ2 subunits in mouse CD4^+^ and CD8^+^ T cells (Fig. 3E). The GABA-A γ2 specific immunostaining in mouse T cells was blocked by pre-absorption of the antibody with γ2 immunogenic peptide (Fig. 3F). In Jurkat cells (Fig. 3D), the immunostaining for the GABA-A α1 and β3 appeared more diffuse throughout the cytoplasm.

**Figure 4:**
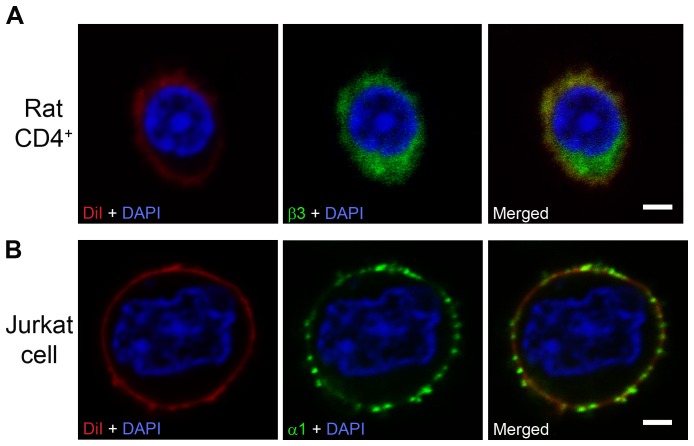
GABA-A channel subunits immunolabeled in the cytoplasm and in the plasma membrane. (A) Rat CD4^+^ T cell, β3 GABA-A channel subunit immunolabeling was observed both in the cytoplasm and in the plasma membrane (n = 41). (B) Jurkat cell, α1 GABA-A subunit immunolabeling was observed prominently in the plasma membrane as punctate pattern (n = 59). Plasma membrane labelling: DiI (red); subunit colour-labeling: β3 (green), α1 (green). The nuclei were stained with DAPI (blue). Scale bar = 2 µM.

In order to examine the cellular location of these GABA-A channel subunits, we co-labeled the membrane of rat CD4^+^ and Jurkat cells with a lipophilic dye (DiI red). The dye labeled the plasma membrane for both cell-types and in some cells, gained entry into the cells (Fig. 4A and B). The β3-antibody and α1-antibody subunit labeling was present in the plasma membrane of rat CD4^+^ and Jurkat cells demonstrating that GABA-A receptors do reach the plasma membrane (Fig. 4A and B).

### GABA-activated currents in T lymphocytes

**Figure 5:**
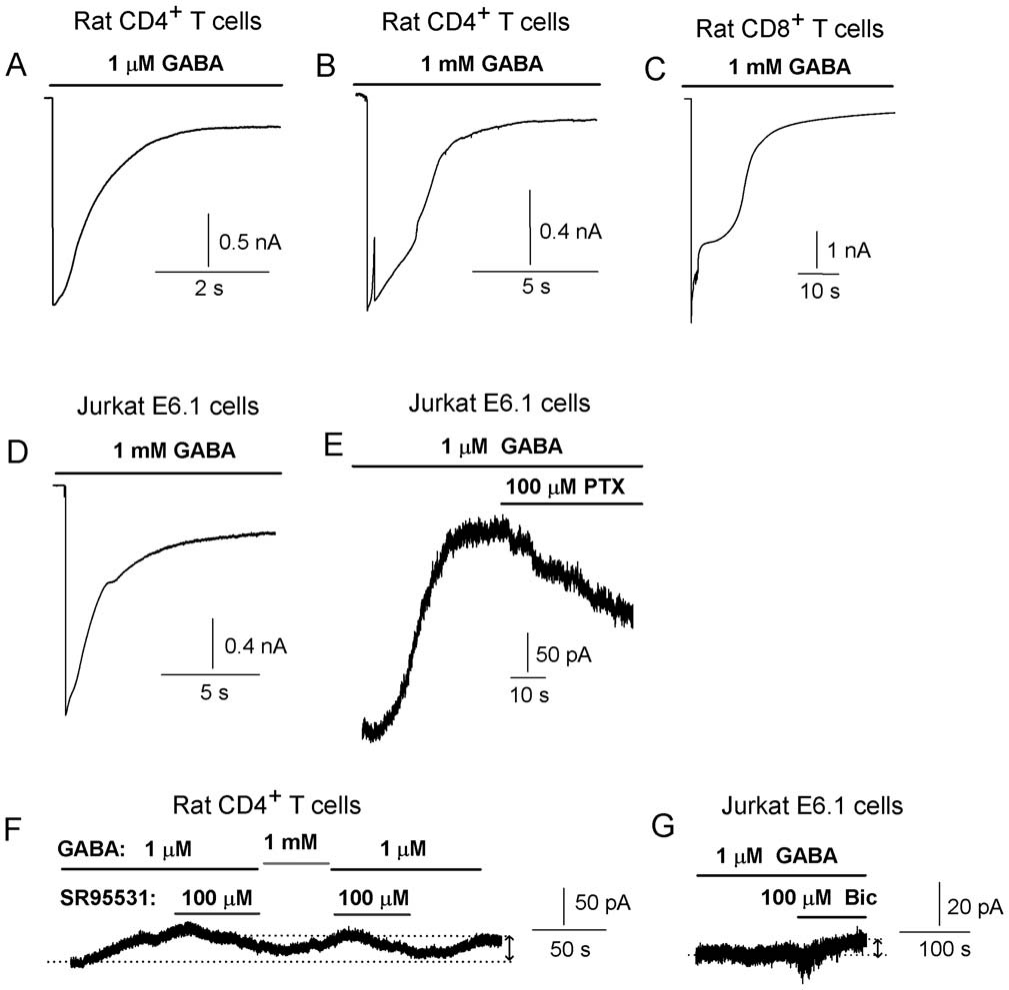
GABA activates GABA-A currents in T cells. Whole-cells currents were evoked by application of 1 µM or 1 mM GABA to rat CD4^+^ T cells (A, B, F), rat CD8^+^ T cells (C) or Jurkat E6. 1 cells (D, E, G). In symmetrical chloride solutions the currents were inward at negative potentials (A, B, C, D; −80 mV and in G, −60 mV) and outward at positive potential (E, F; +40 mV). Picrotoxin (PTX) inhibits the GABA-activated transient current. (F, G) Tonic currents were activated by GABA (1 µM, 1 mM) and inhibited by 100 µM SR95531 (F) or 100 µM bicuculline (G), GABA-A channel antagonists. The difference between the dotted lines shows the amplitude of the tonic current (51 pA). Applications of drugs are indicated by the bars located above the current traces.

We further examined if functional GABA-A channels could be detected in the T cells and Jurkat cells using the patch-clamp technique. We recorded whole-cell GABA-activated transient or tonic currents from rat CD4^+^ (n = 6) and CD8^+^ (n = 5) T cells and Jurkat cells (n = 6) (Fig. 5). At a negative holding potential (−80 mV) in chloride solutions (E_Cl_ = −20 mV), 1 µM or 1 mM GABA application to the cells resulted in an inward, transient current where the peak amplitude ranged from −1.2 to −6.8 nA (n = 7, holding potential = −80 mV, Fig. 5 A–D). Surprisingly, from each cell we often only recorded one current response. Subsequent applications of GABA resulted in no current activation indicating an extensive run-down of the response in the cells. The currents were outward at positive holding potentials and blocked by 100 µM picrotoxin (+40 mV, Fig. 5E, n = 2) or 100 µM bicuculline (n = 1, not shown). Extrasynaptic GABA-A channels generate small amplitude but long-lasting currents in neurons that is termed tonic current. We examined if we could activate tonic currents in the T cells. Tonic currents were activated with 1 µM or 1 mM GABA and inhibited with 100 µM SR95531 (holding potential = +40 mV, Fig. 5F) in rat CD4^+^ (n = 2) and CD8^+^ (n = 3) T cells or 100 µM bicuculline (Jurkat cells, holding potential = −60 mV, n = 2, 5G). The whole-cell current results show that T cells and Jurkat cells express functional GABA-A channels that respond with transient or tonic currents when exposed to GABA.

## Discussion

Our results demonstrate that human, mouse and rat CD4^+^ and CD8^+^ T cells express GABA-A channel mRNAs that are translated into proteins that form functional channels in the plasma membrane of the cells. CD4^+^ and CD8^+^ T cells express the same GABA-A subunits but the specific isoforms differ between the species. Five, eight and thirteen different types of GABA-A subunits were detected in the T cells from humans, C57BL/6J mice and Wistar rats, respectively. Each species expressed at least two α subunit isoforms, one or more types of β subunits but differed widely in what other types of subunits were expressed. The different profile of subunit isoforms expressed in the cells from humans, mice and rats is highly significant as it demonstrates that the GABA-A channel subtypes will vary according to species. The Western blots and immunostaining images demonstrated that the GABA-A channel proteins are abundant and located in the plasmalemma and throughout the cytoplasm of the CD4^+^ and the CD8^+^ T cells. The staining pattern often appeared punctate, possibly indicating clustering of channels in vesicles or the plasma membrane. GABA evoked transient and tonic currents in the cells, somewhat similar to what is recorded in neurons.

There have been relatively few studies that have examined the expression of the GABA-A channels in immune cells [5]. The GABA-A transcripts are often present in the cells but it varies which subunits have been detected. α1, α2, β1, β2, γ3 and δ were identified in CD4^+^ T cells from the type-1 diabetic NOD (non-obese diabetic) mice [11] whereas in CD4^+^ T cells from an experimental autoimmune encephalomyelitis (EAE) mouse model α1, β1, γ2 and ε were examined but not detected [21]. α1, α4, β2, β3, γ1 and δ were detected in an EAE cell line [8] and in CD4^+^ and CD8^+^ T cells from Biobreeding (BB) rats, α1, α2, α3, α4, α6, β3, γ1, δ, ρ1 and ρ2 were identified [18]. In these studies, only in two cases [8], [18] have all 19 subunits been examined. It is, therefore, possible that more subunit isoforms can be detected in the T cells from the mouse models. However, the NOD mouse expressed the γ3 subunit that we did not detect in the C57BL/6J mouse model used in this study and the combination of subunits expressed in the BB rats differ from the Wistar rats in this study. Whether it is the strain of animals or possibly the state of activation of the cells that regulates the subunit isoforms expression pattern remains to be determined. α1, α2, β3 and δ have been detected in cultured peritoneal macrophages and β1 and ε from macrophages isolated from an EAE mouse model [21]. Human peripheral monocytes have been reported to express the α1, α3, α4, β2, β3, δ and ε subunits [6], [22] or only the β2 subunit [13]. Dionisio et al. (2011) also examined human periperal monocytes and consistently detected the α1, δ and ρ2 subunits. Clearly, immune cells from humans, mice and rats do have the necessary building blocks to form GABA-A ion channels but what determines which subtype of the GABA-A channel is expressed and whether the expression varies with the state of the activation of the T cells or even among different subtypes of T cells (e.g. naïve T, T_reg_, T_H_, T_CM_, T_EM_ cells) remains to be clarified.

The physiology and pharmacology of GABA-A channels (GABA-A receptor) is determined by the subunit composition of the channel [1]. In human T cells, the pentameric GABA-A channels may be formed by αβ subunits alone or in combination with the ρ2 and even the π subunits. The αβ GABA-A channel subtype does exist in the brain but represents a minority of neuronal GABA-A channels [14], [23]. The functional and pharmacological properties of the αβ channels have been extensively studied in heterologous expression systems. These channels are sensitive to drugs like barbiturates (pentobarbital [24]), intravenous (propofol, etomidate [25]) and volatile anaesthetics (isoflurane, halothane, chloroform [26]) but not to the benzodiazepines (diazepam, [27], [28]) that require the incorporation of the γ2 subunit in the channel complex [1], [27]. Less is known about channels containing the ρ2 and the π subunits [1]. The ρ2 subunit can also form pentameric channels alone [29], [30]. These homomeric channels have in the past been referred to as the GABA-C channels but today they are considered a subtype of the GABA-A channels [1].

Precaution needs to be taken when selecting model animals for studies. Although, both rats and mice can serve as good models for studying various functional properties of the GABA-A channels in immune cells and how the channels influence the function of the immune system, the GABA-A channel subtypes in humans and rodents CD4^+^ and CD8^+^ T cells differ. The β subunits have limited influence on the pharmacology of the channels whereas the α subunits and the potential 3^rd^-type of subunit in the channel complex, e.g. γ, δ, ρ, can have dominating effects [1]. The expression profile of subunit isoforms is similar for the native human T cells and the Jurkat cell line. Importantly, the highest expressing α subunit is the same (α1) and so are the non-α, β subunits, ρ2 and π. As a model system for examining pharmacological properties of human T cells, the Jurkat cell-line appears to be a possible alternative.

Perhaps the most significant difference among the CD4^+^ and CD8^+^ T cells from humans, mice and rats is that only in mice is the γ2 subunits expressed. As the human GABA-A CD4^+^ and CD8^+^ T cells do not contain the γ2 subunits, those channels will not be modulated by anaesthetics such as diazepam but may be modulated by intravenous and volatile anaesthetics that only require α and β subunits in the channels complex to enhance the GABA-A channel function [1], [25], [26]. Our results support the hypothesis put forth by Wheeler et al. (2011) that side-effects of anaesthetics on immune function via GABA-A channels sometimes observed in intensive care units may potentially be decreased by selecting drugs that do not modulate e.g. GABA-A function in T cells.

GABA can affect a wide variety of functional properties of immune cells like cytokine secretion, cell proliferation, phagocytic activity and chemotaxis [5] but we know relatively little about the mechanism of how GABA influences these processes. In the T cells not only are the GABA-A channels transcripts present but the channel proteins are abundantly expressed and form functional channels. The cells respond with large amplitude, fast decaying currents or low amplitude, long lasting currents when exposed to GABA. These current responses are reminiscent of currents observed in neurons where they regulate neuronal excitability. By modulating the membrane potential, the GABA-A channels in T cells may affect a number of processes that take place in the cells. Changes in the membrane potential will affect the open probability of other channels present in the plasma membrane such as voltage-gated Ca^2+^ channels. A shift in the membrane potential also affects the driving force on ions crossing the cell membrane though specific channels such as the CRAC (Ca^2+^ release-activated Ca^2+^) channel. These processes will influence e.g. the intracellular calcium concentration.

In conclusion, our study demonstrates the presence of multiple, functional GABA-A channels in CD4^+^ and CD8^+^ T cells from humans, mice and rats. The subtype profile differs between the species. It is still not known whether these interspecies differences in T cells also exist in other subpopulation of immune cells. Depending on the subunit composition of GABA-A channels in the immune cell, the response to drugs acting at GABA-A channels may vary widely and therefore differentially influence the immune cells. This is important to bear in mind when selecting the appropriate animal models to study the role of GABA and the GABA-A channels in the immune cells.

## Materials and Methods

### Ethics Statement

#### Animal and Human Tissue

Wistar rats and C57BL/6J mice at the age of 65 days and 142–146 days, respectively, of both sexes were used in the study. All animals were housed and bred in specific pathogen free conditions. Animals were kept in 12 h light and 12 h dark cycle with pellet food and water. The animals were sacrificed in accordance with local ethical guidelines, and the animal care protocols (approval number C244/11) were specifically approved by the Uppsala Djurförsökiska Nämd, Sweden (the animal ethics committee for Uppsala University). Adipose tissue containing lymph nodes from cadaver donors in Ringer solution was obtained from the Nordic Islet Transplantation Program (www.nordicislets.org) by the courtesy of prof. Olle Korsgren, Uppsala University. The research program and written informed consent for the original human work that produced the tissue samples were approved of by regional etikprövningsnämnden Uppsala (the ethics committee for Uppsala University) and was given the approval number 2009/298.

#### Tissue Collection and Cell Isolation Procedure

The procedure has been described in detail [18]. In brief, mesenteric lymph nodes were collected from rats or mice and placed in ice-cold 1× PBS (phosphate buffered saline). The lymph nodes were then minced to release the cells. The tissue mix was filtered and a pure single cell suspension obtained. Human pancreatic lymph nodes were collected from pancreatic adipose tissue from deceased human donors and followed the same procedure to get the single cell suspension.

#### Separation of rat, mouse and human CD4+ and CD8+ T cell

To separate rat or mouse CD4^+^and CD8^+^ T cells, the cell suspensions were labeled and incubated for 15 min at 4–8°C with rat CD8a or CD4 Microbeads or mouse CD8a(ly-2) or CD4 (L3T4) Microbeads (Miltenyi Biotec, Germany), respectively. To separate human CD4^+^ and CD8^+^ T cells, the cell suspensions were labeled and incubated for 10 min at 4–8°C with primary non-CD8^+^ or non-CD4^+^ T cell biotin antibodies cocktail, and further incubated with secondary anti-biotin Microbeads for additional 15 min at 4–8°C. The respective labeled cells were washed with MACS buffer containing PBS pH 7.2, 2 mM EDTA, 0.5% BSA (MiltenyiBiotec, Germany) and centrifuged for 10 min at 300× *g* at 4°C. The cell pellets were resuspended in MACS buffer. The magnetically labeled cells suspended in the MACS buffer were separated by using manual MACS cell separation set-up with LS columns and magnet (Miltenyi Biotech, Germany). The positive labeled cells of rat or mouse CD4^+^ and CD8^+^ T cell fractions were collected independently. The negative selection protocol was used to separate human CD4^+^ and CD8^+^ T cell fractions. The separated CD4^+^ and CD8^+^ T cells were assessed for viability using trypan blue exclusion method and counted.

#### Cell Line Cultures

Jurkat E6. 1 cells (human leukaemic T cell lymphoblast) obtained from ECACC (Salisbury, UK) were seeded in RPMI-1640 (2 mM glutamine, 25 mM HEPES, 10% heat inactivated FBS, 100 U/ml penicillin, 10 µg/ml streptomycin, 5 µM β-mercaptoethanol) at a density of 5×10^5^ cells/ml and every two days the cells grew to 80% confluence. Once confluent Jurkat cells were collected for experiments.

### RNA isolation and RT-qPCR

The methods have been described in detail [18], [31]. Briefly, total RNA was extracted from the magnetically separated CD4^+^ and CD8^+^ T cells from rat, mice, humans and Jurkat E6. 1 cell line using the GenElute total RNA miniprep (Sigma). The gene-specific primer pairs (primer sequences shown in Table 1) were designed using NCBI's Primer-BLAST or Primer Express Software version 3.0 (Applied Biosystems) and further verified using the appropriate species brain cDNA. SYBR Green chemistry was used to detect target genes. Reference genes were selected from commonly used “housekeeping” genes. In humans we examined beta-actin (ACTB), beta-2-microglobulin (B2M), 18S rRNA (eukaryotic 18S ribosomal RNA), glyceraldehyde-3-phosphate dehydrogenase (GAPDH) and selected B2M in accordance with the literature [32], [33], [34]. In rats we then examined the level of expression of GAPDH, hypoxanthinephosphoribosyl-transferase (HPRT), cyclophilin A (PPIA), 18S rRNA and selected the HPRT based on appropriate level of expression and minimal variability [35], [36], [37]. In mouse we used ACTB [34], [38].

### Western Blot Analysis

For extraction of proteins from T cells and Jurkat cells, cell lysis buffer [12.5 mM Tris base, 12.5 mM NaCl, 1% Triton X-100 and protease inhibitor cocktail tablet (Roche Applied Science), pH 7.4] was used while brain proteins were extracted by homogenizing total brain in tissue homogenization buffer (12.5 mM Tris base, 37.5 mM NaCl, 1 mM MgCl_2_, 0.125 mM EDTA, 2% Triton X-100 and protease inhibitor cocktail tablet, pH 7.4). The sample was incubated for 10–20 min at 4°C, homogenized with a sterile pestle, and then passed through a syringe twice followed by centrifugation at 15000× g for 20 min. The supernatant was collected and the protein concentration was measured in System multiscan instrument using the RC DCTM protein assay kit (Bio-Rad, USA). Protein samples (10–60 g) subjected to SDS-PAGE using 10% polyacrylamide gels and transferred to PVDF membranes (Amersham Biosciences). The membranes were blocked with 5% fat-free milk in Tris buffered saline containing 0.1% Tween (TBS-T) for 1 h and incubated overnight at 4°C with the following primary antibodies: rabbit anti-GABA_A_R α1–6 (1∶200, Santa Cruz Biotechnology, Germany), rabbit anti-GABA_A_R α1 (1∶1000, Synaptic Systems, Germany), goat anti-GABA_A_R β3 (1∶500, Santa Cruz Biotechnology, Germany), rabbit anti-GABA_A_R γ2 (1∶500, Synaptic Systems, Germany) and rabbit anti-β-actin (1∶1000, Biovision, USA), respectively. Experiments using the γ2 blocking-peptide for rabbit anti-GABA_A_R γ2 (Synaptic Systems, Germany) were done a similar way except that the γ2 antibody was pre-incubated with 10 fold higher concentration of the γ2 peptide for 1 h. After washing with TBS-T, the membranes were further incubated with horseradish peroxidase-conjugated secondary antibody (1∶10000, Jackson Immunoresearch laboratories, USA) for 1 h and then the immunoreactive protein bands were visualized by enhanced chemiluminescence (ECL) detection kit (GE Healthcare, Sweden).

### Immunocytochemistry and Confocal Imaging

Cells were resuspended in PBS and stained either in Falcon tubes or on poly-L-lysine or TESPA (3-triethoxysilylpropylamine) coated cover slips. Cells were fixed with 4% paraformaldehyde (PFA) in 0.1 M phosphate buffered saline (PBS) for 20 min at RT. After fixation, cells were washed with PBS, blocked with 5% bovine serum albumin (BSA) in PBS for 15 min and incubated overnight at 4°C with the following primary antibodies: rabbit anti-GABA_A_R α1 (1∶50, Novus Biologicals, USA) or rabbit anti-GABA_A_R α1 (1∶500, Synaptic Systems, Germany), rabbit anti-GABA_A_R α2 (1∶500, Synaptic Systems, Germany), rabbit anti-GABA_A_R γ2 (1∶500, Synaptic Systems, Germany) and mouse anti-GABA_A_R β3 (1∶500, NeuroMab, USA), respectively. The control experiment for the γ2 antibody was performed in the mouse CD4^+^ T cells in the same way as with the γ2 antibody apart from pre-incubation with 10 fold higher concentration of the γ2 blocking peptide for 1 h. After washing with PBS, cells were incubated with a fluorophore-conjugated secondary antibody (Jackson Immunoresearch Laboratories, USA) at RT for 1 h. All antibodies were diluted in 5% donkey serum in PBS. Cell nuclei were stained with DAPI and the plasma membrane was labeled with lipophilic tracer DiI (Invitrogen, USA) as indicated. The cells were immersed in the fluorescent mounting media (Dako, Sweden), and examined with a confocal microscope (LSM Meta, Carl Zeiss, Germany). The omission of primary antibodies served as negative controls.

### Electrophysiological Recording

The isolated rat CD4^+^ and CD8^+^ T cells or Jurkat cells were collected (1–3×10^6^ cells) and centrifuged for 2 min at 100× g. The supernatant was removed and the cell pellet was washed with the extracellular solution containing in mM: 145 NaCl, 5 KCl, 1 MgCl_2_, 1.8 CaCl_2_, 10 TES pH 7.3, 297 mOsm, centrifuged for 2 min at 100× g and then resuspended in 50–100 µl extracellular solution. Nanion's Port-a-Patch chip technology (Nanion, Germany) or conventional patch-clamp methods were used to patch-clamp the cells. Using the Port-a-Patch chip technology the success rate of obtaining whole-cell recordings of native T cells was greater than with the conventional-patch-clamp method. For the Port-a-Patch, a 5 µl cell suspension (10^6^ cell/ml) was dispensed into the extracellular chamber containing the recording chip, resistance 5 or 8–10 MΩ for rat T cells and 2–3 or 5 MΩ for Jurkat cells. The whole-cell configuration was established and currents were recorded at holding potentials of −80 mV or + 40 mV. For the conventional patch-clamp recordings, the holding potential was −60 or + 40 mV. GABA (1 µM or 1 mM) and GABA plus 100 µM SR95531, 100 µM bicuculline or 100 µM picrotoxin (all drugs from Sigma, Germany) were prepared in the extracellular recording solution and perfused into the extracellular chamber at a rate of 1 ml/min. The Port-a-Patch internal recording solution contained in mM: 50 CsCl, 10 NaCl, 60 Cs-fluoride, 20 EGTA, 10 mM TES pH 7.3, 284 mOsm. The extracellular recording solution contained in mM: 80 NaCl, 3 KCl, 10 MgCl_2_, 35 CaCl_2_, 10 HEPES pH 7.3, 296 mOsm. The conventional patch-clamp pipette (internal) solution contained in mM: KCl 135, CsCl 5, CaCl2 1.8, MgCl2, TES 15 pH 7.4 282 mOsm and the extracellular solution contained in mM: NaCl 145, KCl 5, MgCl2 1, CaCl2 1.8 Ph 7.3, 297 mOsm. 5 mM EGTA was sometimes included in the pipette solution. All patch-clamp recordings were performed at room temperature (20–22°C). Currents were recorded using an Axopatch 200B amplifier, filtered at 2 kHz, digitized on-line at 10 kHz using an analogue-to-digital converter and analyzed with pClamp 10.2 software (Molecular Devices, USA).

### Statistical analyses

Data are presented as mean ± SEM. Differences in expression levels were analyzed using one way ANOVA or Kruskal-Wallis one way ANOVA on ranks, post hoc Tukey test. All statistical tests were performed using Sigma Plot version 11 (Systat Software, San Jose, CA, USA).

## Supporting Information

Table S1 **Molecular weight of GABA-A channel subunits.** Values were calculated from the amino acid sequences as given in the NCBI data base.
(DOC)
