# Supplementary material for: Different Subtypes of GABA-A Receptors Are Expressed in Human, Mouse and Rat T Lymphocytes
Source: PLoS One. 2012 Aug 21;7(8):e42959. doi: 10.1371/journal.pone.0042959 (PMC3424250; doi:10.1371/journal.pone.0042959)
Supplement: Table S1 — Molecular weight of GABA-A channel subunits. Values were calculated from the amino acid sequences as given in the NCBI data base. (DOC) [file pone.0042959.s001.doc]

Table S1. Molecular weight of GABA-A channel subunits.

| GABA-A Subunit | Rat  (kDa) | Mouse  (kda) | Human  (kDa) |
| --- | --- | --- | --- |
| α1 | 51.76 | 51.76 | 51.80 |
| α1 | 54.07 | 54.10 | 54.23 |
| α2 | 51.26 | 51.14 | 51.33 |
| α2 | 54.63 | 54.63 | 54.61 |
| α3 | 55.43 | 55.40 | 55.15 |
| α3 | 54.18 | 54.28 | 54.11 |
| α4 | 60.95 | 60.88 | 61.62 |
| α5 | 52.41 | 52.27 | 52.15 |
| α6 | 51.18 | 51.10 | 51.02 |
| β1 | 54.07 | 54.10 | 54.23 |
| β2 | 54.63 | 54.63 | 54.61 |
| β3 | 54.18 | 54.28 | 54.11 |
| γ1 | 53.55 | 53.41 | 53.60 |
| γ2 | 55.1 | 55.13 | 54.16 |
| γ3 | 54.29 | 54.33 | 54.29 |
| δ | 50.57 |  |  |
| ε | 59.22 |  |  |
| θ | 72.57 |  |  |
| π | 50.48 |  |  |
| ρ1 | 54.85 |  |  |
| ρ2 | 54.30 |  |  |
| ρ3 | 54.03 |  |  |

Values were calculated from the amino acid sequences as given in the NCBI data base..
